# It’s What We Do: Māori Carers Talk About Roles, Challenges, and Coordination in Caring for Older Family Members

**DOI:** 10.1093/geroni/igaa057.1641

**Published:** 2020-12-16

**Authors:** Mary Simpson, Kirstie McAllum, Christine Unson, Stéphanie Fox, Te Oha Hancock

**Affiliations:** 1 University of Waikato, Hamilton, Waikato, New Zealand; 2 Universite de Montreal, Montreal, Canada; 3 Southern Connecticut State University, New Haven, Connecticut, United States; 4 University of Waikato, Hamilton, New Zealand

## Abstract

As the population of Aotearoa/New Zealand heads towards one-in-four being aged over 65-years-and-over by 2040, it is anticipated that family members will play an increasingly important role in caring for older relatives with chronic and age-related health issues. Multi-generational, and in particular three-generational living arrangements, combined with family care of older relatives are a growing trend; a trend already even evident among Māori communities. This paper reports on a study that explored the care experiences and expectations of 14 past and current Māori carers (aged 23 to 72-years) of older relatives. Interviews were audio-recorded, transcribed verbatim, and coded independently. The initial thematic analysis revealed nine themes and participants were invited to feedback on the summary in person or in writing. The feedback resulted in the original themes being collapsed into four (with subthemes): “Care is Normal”; “Collective Coordination of Care”; “Insider-Carer—Outsider Perspectives on Caring”; and “Societal Influences on Family Care/Carers”. Firstly, these themes highlight how Māori cultural norms infuse direct care, support, and coordination roles within family care of the older family member. Secondly, they reveal the challenges for family carers in talking about their work with others, especially (thirdly) in the face of negative attitudes towards care and carers of an older family member within wider society. These findings have implications for cultural and wider socio-political influences in socialising different groups to carer role expectations. If society is to better prepare future caregivers for their role, ongoing research is needed with the various cultural groups in Aotearoa/New Zealand.

